# Differential Activity of the Combination of Vancomycin and Amikacin on Planktonic vs. Biofilm-Growing *Staphylococcus aureus* Bacteria in a Hollow Fiber Infection Model

**DOI:** 10.3389/fmicb.2018.00572

**Published:** 2018-03-27

**Authors:** Diane C. Broussou, Marlène Z. Lacroix, Pierre-Louis Toutain, Frédérique Woehrlé, Farid El Garch, Alain Bousquet-Melou, Aude A. Ferran

**Affiliations:** ^1^INTHERES, INRA, ENVT, Université de Toulouse, Toulouse, France; ^2^Vétoquinol, Global Drug Development, Lure, France; ^3^Department of Veterinary Basic Sciences, Royal Veterinary College, London, United Kingdom

**Keywords:** hollow-fiber infection model, antibiotic combination, amikacin, vancomycin, biofilm, antimicrobial resistance, *Staphylococcus aureus*

## Abstract

Combining currently available antibiotics to optimize their use is a promising strategy to reduce treatment failures against biofilm-associated infections. Nevertheless, most assays of such combinations have been performed *in vitro* on planktonic bacteria exposed to constant concentrations of antibiotics over only 24 h and the synergistic effects obtained under these conditions do not necessarily predict the behavior of chronic clinical infections associated with biofilms. To improve the predictivity of *in vitro* combination assays for bacterial biofilms, we first adapted a previously described Hollow-fiber (HF) infection model by allowing a *Staphylococcus aureus* biofilm to form before drug exposure. We then mimicked different concentration profiles of amikacin and vancomycin, similar to the free plasma concentration profiles that would be observed in patients treated daily over 5 days. We assessed the ability of the two drugs, alone or in combination, to reduce planktonic and biofilm-embedded bacterial populations, and to prevent the selection of resistance within these populations. Although neither amikacin nor vancomycin exhibited any bactericidal activity on *S. aureus* in monotherapy, the combination had a synergistic effect and significantly reduced the planktonic bacterial population by -3.0 to -6.0 log_10_ CFU/mL. In parallel, no obvious advantage of the combination, as compared to amikacin alone, was demonstrated on biofilm-embedded bacteria for which the addition of vancomycin to amikacin only conferred a further maximum reduction of 0.3 log_10_ CFU/mL. No resistance to vancomycin was ever found whereas a few bacteria less-susceptible to amikacin were systematically detected before treatment. These resistant bacteria, which were rapidly amplified by exposure to amikacin alone, could be maintained at a low level in the biofilm population and even suppressed in the planktonic population by adding vancomycin. In conclusion, by adapting the HF model, we were able to demonstrate the different bactericidal activities of the vancomycin and amikacin combination on planktonic and biofilm-embedded bacterial populations, suggesting that, for biofilm-associated infections, the efficacy of this combination would not be much greater than with amikacin monotherapy. However, adding vancomycin could reduce possible resistance to amikacin and provide a relevant strategy to prevent the selection of antibiotic-resistant bacteria during treatments.

## Introduction

*Staphylococcus aureus* possesses the ability to form biofilms and is responsible for chronic infections which are hard to treat and cause significant morbidity and mortality.

Biofilms are communities of bacteria which adhere to surfaces and are encapsulated in a self-produced extracellular polysaccharide matrix. They constitute an important strategy implemented by microorganisms to survive in harsh environmental conditions (Donlan and Costerton, 2002). Biofilms are responsible for chronic, recurrent infections and are known to survive very high concentrations of antibiotics (Lewis, 2008; Lebeaux et al., 2014). One hypothesis to explain the lower activity of antimicrobial drugs on biofilms is the high prevalence of persister cells in biofilms (Lewis, 2008; Singh et al., 2009). These persisters, unlike resistant bacteria which are genetically modified, consist of clones of bacteria expressing a different but reversible phenotype which allows them to transiently escape the effects of antibiotics (Lewis, 2008).

The antibiotic therapies currently used against biofilm infections are often associated with poor clinical responses and frequent relapses (Davies, 2003). For several years, different solutions have been proposed to eradicate biofilm bacteria such as phages, quorum sensing inhibitors or physical methods (Ivanova et al., 2017). However, although highly innovative strategies still need to be developed to deal with severe infections by both tolerant and multi-resistant bacteria, the method which can most rapidly and easily be implemented in patients at present is to combine existing drugs or to modify their therapeutic regimen (dose, frequency, and mode of administration).

In the case of suspected *S. aureus* infection, vancomycin therapy is often initiated in patients to provide antibacterial activity against both Methicillin-Sensitive *S. aureus* (MSSA) and Methicillin-Resistant *S. aureus* (MRSA) (Deresinski, 2009). However, although vancomycin can kill planktonic bacteria, its activity against Biofilm-Embedded Bacteria (BEB) is quite low. Lebeaux et al. (2015) showed that after exposure to a very high, constant concentration of vancomycin (5000 mg/L) for 24 h, the percentage of bacteria surviving in a 24 h-old *S. aureus* biofilm exceeded 20% and was even close to 100% for 2 of the 4 tested strains. Singh et al. (2009) reported similar results and found no statistically significant difference between the bacteria remaining in a non-treated *S. aureus* biofilm or in a biofilm exposed for 24 h to vancomycin concentrations equal to or higher than those clinically achievable. Post et al. (2017) demonstrated that vancomycin is able to eradicate a mature biofilm of *S. aureus* from metal implants by using a static concentration of 200 mg/L over 28 days. Nevertheless, such a concentration profile cannot be achieved by systemic administration or local delivery vehicles currently available. To overcome this poor activity on biofilms, an aminoglycoside is often combined with vancomycin. Synergistic activity between vancomycin and aminoglycosides had already been demonstrated on *S. aureus* (Watanakunakorn and Glotzbecker, 1974; Cokça et al., 1998) but these studies were performed by exposing planktonic bacteria for no more than 24 h to constant antibiotic concentrations whereas in the *in vivo* situation, antibiotic concentrations continuously fluctuate over several days. The effects of a combination of gentamicin and vancomycin on *S. aureus* were more rarely tested under dynamic *in vitro* conditions with varying antibiotic concentrations or in animal models of infection. No significant synergy was observed in two studies where low inocula of *S. aureus* were exposed to the two drugs (Backo et al., 1999; Aeschlimann et al., 2000). Another study on large inocula of MRSA and MSSA, representative of a biofilm- associated infection, was performed in an *in vitro* simulated endocardial vegetation model. The effect of vancomycin alone was statistically significant compared to the control after 3 days but the activity of vancomycin on MSSA or MRSA was not improved by adding gentamicin (LaPlante and Woodmansee, 2009). However, in this study, the vancomycin concentrations tested were almost two times higher than the free and active concentrations routinely obtained in patients because no correction was performed for the 45% plasma protein binding of vancomycin (Liu et al., 2002; Butterfield et al., 2011).

To propose new treatment optimizations, the predictivity of *in vitro* experiments needs to be improved, for example by exposing both planktonic and BEB in parallel over the complete duration of treatment (several days), to drug concentrations identical to those that would be encountered under clinical conditions in patients.

In this study, we studied the effects of amikacin, an aminoglycoside, and vancomycin on planktonic and biofilm-embedded *S. aureus* by using an *in vitro* dynamic model, the Hollow-Fiber (HF) infection model, which mimics the fluctuations of antibiotic concentrations over time, as would occur in the plasma of patients during a 5-day treatment. The HF model was recently labeled by the European Medicines Agency (European Medicines Agency, 2015; Gumbo et al., 2015) for drug dosage optimization in the treatment of tuberculosis. We have further adapted this model to explore drug activity not only on planktonic but also on biofilm-embedded *S. aureus*. Indeed, in previous studies conducted in HF (Nicasio et al., 2012; Ferro et al., 2015), the bacteria were systematically exposed to drugs during the exponential phase of growth, when there was no time for biofilm development, whereas in this study, the biofilm was allowed to form for 3 days before drug exposure. The killing effects of drugs and the potential selection of resistance were assessed both on planktonic bacteria over time and on BEB at the end of exposure. We first compared monotherapy and combinations of amikacin and vancomycin at the currently recommended dosing regimens, i.e., 1g vancomycin twice a day and 15 mg/kg amikacin once a day for 5 days. Such therapeutic regimens are considered sufficient to achieve the PK/PD indices classically expected to obtain drug efficacy. For aminoglycosides, the most predictive PK/PD index is the Maximal Concentration (*C*_max_) divided by the Minimal Inhibitory Concentration (MIC) ratio (Moore et al., 1987) and a value from 8 to 10 is usually recommended to ensure efficacy against the pathogen (Toutain et al., 2002). For vancomycin, the best predictive index is the AUC over 24 h divided by the MIC (AUC_24h_/MIC) (Nielsen et al., 2011), and value of 400 is recommended to achieve clinical effectiveness (Rybak et al., 2009; Jung et al., 2014; Song et al., 2015).

We then explored the effects of a slight deviation from these standard dosages by simulating an increased dose of amikacin, which is a concentration-dependent antibiotic, (Frimodt-Møller, 2002) and by modifying the mode of administration (infusion vs. bolus) of vancomycin, which is a time-dependent antibiotic (Waineo et al., 2015).

## Materials and Methods

### Bacterial Strain

The Methicillin-sensitive *S. aureus* strain HG 001, derived from NCTC 8325, was used for all experiments.

### Antimicrobial Agents

Amikacin sulfate powder (Amikacine Mylan^®^) and vancomycin chlorhydrate powder (Vancomycine Sandoz^®^) were used to prepare antibiotic stock solutions with water. Vials were stored at -20°C for less than 1 month and were thawed and diluted to the desired concentrations for the assay just before each antibiotic administration.

### Minimal Inhibitory Concentration (MIC) Determination

Minimal inhibitory concentrations of vancomycin and amikacin on the MSSA strain were performed in triplicate by broth microdilution in cation-adjusted Mueller Hinton broth (Ca-MH, Mueller-Hinton II, Sigma Aldrich, Saint Quentin-Fallavier, France) according to the CLSI reference methods (Clinical and Laboratory Standards Institute [CLSI], 2012), and also in Roswell Park Medium Institute 1640 Medium (RPMI, Gibco, Thermo Fischer Scientific, MA, United States). Briefly, a bacterial suspension, diluted in Mueller-Hinton Broth or RPMI to give a final organism density of 5.7 log_10_ CFU/mL, was added to wells of a microtiter plate containing serial twofold dilutions of vancomycin or amikacin. Growth was recorded after incubation for 18 h at 35°C.

### PK/PD Study

#### Hollow-Fiber Infection Model

**FIGURE 1:**
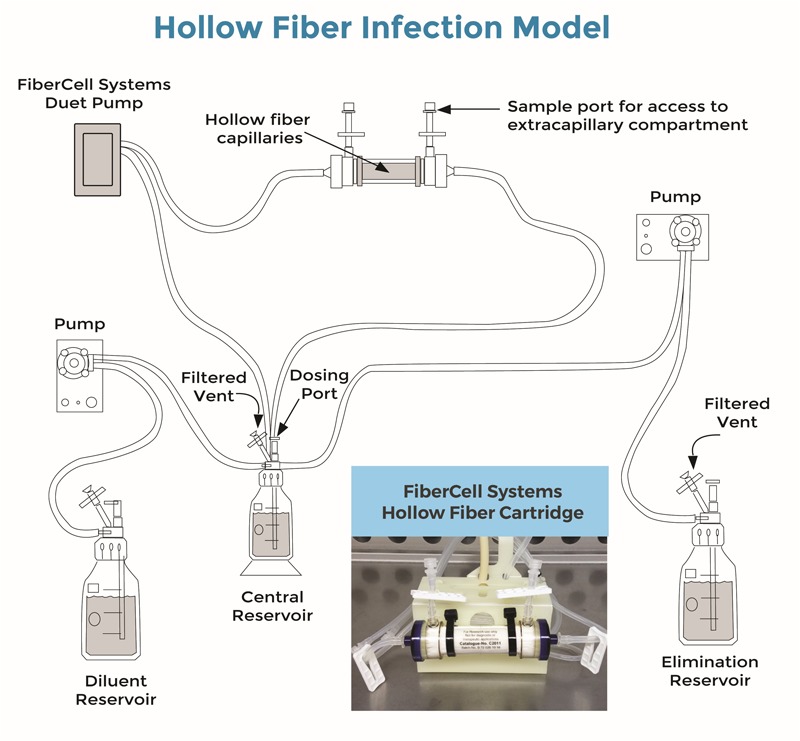
Diagrammatic representation of the Hollow Fiber Infection Model kindly provided by FiberCell Systems^®^ (Cadwell, 2015). Bacteria were trapped by the hollow fiber capillaries in the cartridge (see also embedded photo). Drugs were added to the central reservoir and freely circulated through the cartridge and bacteria by means of the Fibercell Systems Duet pump^®^ (FiberCell Systems, Inc., Frederick, MD, United States). Drug concentrations decreased over time after drug administrations, due to the continuous addition of a diluent (RPMI) by means of another set of pumps (here, Mini Rythmic^®^ PN+, SMD, Fleury-sur-Orne, France).

A HF infection model was used to assess the antibacterial activity of the combination of amikacin and vancomycin on planktonic and biofilm-embedded *S. aureus* during exposure to fluctuating clinically relevant antibiotic concentrations. A diagrammatic representation of the Hollow Fiber Infection Model was kindly provided by FiberCell Systems^®^ (**Figure 1**). Basically, the HF model includes a cartridge with capillaries composed of a semipermeable polysulfone membrane. The pore size of the capillaries (42 kDa) allows equilibration of the concentrations of chemicals which circulate through the central and peripheral compartments by means of a peristaltic pump (Duet pump, FiberCell Systems, Inc., Frederick, MD, United States) while the bacteria stay confined to the extracapillary space in the peripheral compartment.

In this study, twenty milliliters of a suspension containing 5.7 log_10_ CFU/mL of *S. aureus* were inoculated into the extracapillary space of each hollow-fiber cartridge (C2011 polysulfone cartridge, FiberCell Systems, Inc., Frederick, MD, United States) and incubated at 37°C in RPMI from Day 0 (D0) to Day 2 (D2) without any drug, to allow biofilm formation.

From D3 to D7, the bacteria were then subjected to amikacin and/or vancomycin. The drugs were added to the central compartment to obtain the maximum concentration (*C*_max_) and were continuously diluted with RPMI by means of a peristaltic pump (Mini Rythmic PN+, SMD, Fleury-sur-Orne, France) to mimic the human terminal half-life of each antibiotic. The antibiotics also constantly circulated through the central and peripheral compartments by means of a second peristaltic pump (Duet pump, FiberCell Systems, Inc., Frederick, MD, United States).

The first antibiotic exposure tested in the HF model simulated the plasma concentrations of patients receiving 15 mg/kg amikacin once a day (Kato et al., 2017) and/or 1 g vancomycin every 12 h (Nicasio et al., 2012). Since the free plasma drug concentration is known to be one of the best surrogates of the concentration at the site of infection (Liu et al., 2002), we exposed the bacteria in the HF model to concentrations similar to the free plasma concentrations obtained in patients after administration of the above dosing regimens. For amikacin, plasma protein binding was considered negligible and a plasma *C*_max_ of treated patients ranging from 60 to 80 mg/L (A70 treatment) was reproduced in the HF model (Gálvez et al., 2011). For vancomycin, plasma protein binding is around 45% (Butterfield et al., 2011) so the total plasma concentrations obtained from patients described in the literature were corrected to calculate the free *C*_max_ of 18 μg/mL, which was then simulated in the HF model (V18 treatment) (Mandell et al., 2007). The simulated elimination half-life for both drugs in the HF model (4 h) was similar to the plasma elimination half-lives of amikacin and vancomycin in patients (Matzke et al., 1986; Adamis et al., 2004).

For the combinations, we first tested both drugs at the current dosing regimens for amikacin and vancomycin (A70 V18 treatment) and then simulated different pharmacokinetic profiles. We then tested two higher peak concentrations of 90 μg/mL (A90 V18 treatment) and 130 μg/mL (A130 V18 treatment) of amikacin, that could theoretically be attained in patients with a dose of 2500 mg (Álvarez et al., 2016), to investigate the relation between amikacin concentration and activity. For vancomycin, a dosage of 2 g a day has been recently recommended (Patel et al., 2011; Waineo et al., 2015), so a Continuous Rate Infusion (CRI) of 2 g a day of vancomycin was simulated by directly adding the drug to the fresh diluting medium to obtain a constant vancomycin concentration of 9 μg/mL (A70 CRIV9 treatment) (Hanrahan et al., 2015). All the experiments, including control and exposure to amikacin and vancomycin in monotherapy or in combination, were performed in duplicate to check reproducibility.

#### Planktonic Bacteria Quantification

One milliliter samples were collected from the extracapillary space in the HF cartridge to count the planktonic bacteria at 0 h (baseline), 2, 4, 6, 8, and 10 h after the morning antibiotic administration each day for 5 days (D3 to D7). The samples were centrifuged at 3000 *g* for 10 min. The supernatant was removed and the pellet resuspended in 1 mL of NaCl 0.9%. The suspension was then serially diluted and the bacteria counted in triplicate after an overnight incubation at 37°C on tryptic soy agar supplemented with magnesium sulfate and activated charcoal to prevent any carry-over effect of the antibiotic. The counts were verified again 8 h after to include colonies that could have slower grown. The limit of detection was 2.5 log_10_ CFU/mL.

After two washes to remove the antibiotic contained in the suspension, the less-susceptible planktonic bacteria were counted once a day prior to morning antibiotic administrations from D3 to D7 on agars containing threefold (3 μg/mL) and sixfold MIC (6 μg/mL) of amikacin or vancomycin. The plates were incubated for 3 days at 37°C before the bacteria were counted. The proportion of less-susceptible bacteria in the total bacterial population was calculated as the ratio of the colony counts on drug-supplemented agar divided by the colony counts on drug-free agar at the same sampling time.

#### Biofilm Bacteria Quantification

At the end of the experiment (D7), the extracapillary space in the cartridge containing the bacteria was washed four times with 50 mL of sterile NaCl 0.9% to remove the planktonic bacteria. The biofilm was then disrupted by sonication of the cartridge for 15 min at 42 kHz (Bransonic 5800, Branson Ultrasonics Corporation, Emerson, Angoulème, France) which suspended the BEB in the 20 ml of NaCl 0.9% remaining in the cartridge after the washes. These bacteria were collected for quantification with the same technic as for planktonic bacteria. The colonies were plated on the drug-free and drug-supplemented agar and were counted, before and after ultrasound treatment. After an overnight incubation at 37°C, or more if needed, the size of the biofilm was calculated in log_10_ CFU/mL from the difference between the bacterial counts in the extracapillary space before and after ultrasound treatment. For each combination, the MIC of amikacin or vancomycin was also determined on a single bacterial colony growing on the drug-containing agar plates to accurately quantify the loss of susceptibility.

### Drug Assay

Samples for antibiotic quantification were withdrawn from the central reservoir and from the extracapillary space of the cartridge before and after each antibiotic administration and at 2, 4, 6, and 8 h on the 1st day and twice a day thereafter. Samples were centrifuged at 3000 *g* for 10 min and stored at -20°C for less than 2 months before dosing.

Samples were prepared in 1.5 mL tubes. Two hundred μL of 15% of trichloroacetic acid containing the vancomycin d12 and amikacin d5 internal standards at 10 μg/mL were added to 100 μL of calibrators, quality controls, or samples. Antibiotics were quantified on an Acquity ultra performance liquid chromatography (UPLC) coupled to a Xevo triple quadrupole mass spectrometer (Waters, Milford, MA, United States). Chromatographic data were monitored by Targetlynx software (Waters, Milford, MA, United States). The method was validated in terms of linearity, sensitivity and repeatability. Accuracies ranged from 84 to 94% and from 99 to 107% with CV intra-day precisions below 9 and 10% for amikacin and vancomycin, respectively. The limit of quantification was set at 0.5 μg/mL for both antibiotics.

The concentration of antibiotic in the system was calculated according to equation 1.

### Statistics

The planktonic bacterial inoculum sizes before (D3) and after (D7) in the 5-day combined treatments were compared by applying a paired T-test with the R^®^ software (R Development Core Team, 2014).

The sizes of the planktonic bacteria and BEB populations after treatment with the amikacin and vancomycin combination for 5 days (D7) were also compared by paired T-test with R^®^.

## Results

### Minimal Inhibitory Concentration (MIC)

The MIC of vancomycin, for the *S. aureus* strain tested, was 1 μg/mL both in Ca-MH and in RPMI and the MIC of amikacin was 1 μg/mL in Ca-MH and 0.5 μg/mL in RPMI. Based on the EUCAST breakpoints, the tested strain was therefore considered as susceptible to vancomycin and amikacin.

### PK Analysis

**FIGURE 2:**
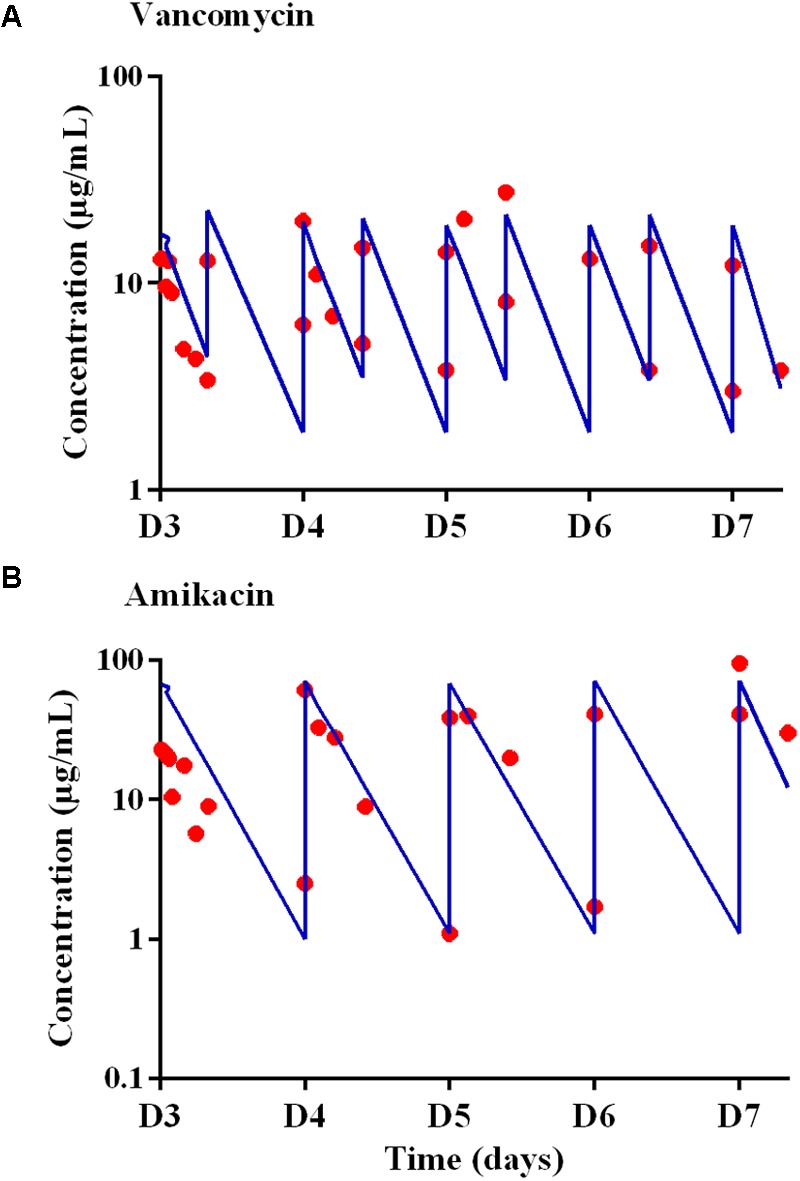
Expected (blue lines) and observed (red circles) concentration-time profiles in the Hollow Fiber system from D3 to D7 for **(A)** vancomycin after administrations twice a day with peak concentrations of 18 μg/mL (V18 treatment) and for **(B)** amikacin after administrations once a day with peak concentrations of 70 μg/mL.

The concentrations in the central compartment and in the extra capillary space of the cartridge (containing bacteria) attained equilibrium within 15 min after adding the antibiotic to the central compartment (data not shown). The predicted vs. observed free concentration-time profiles of amikacin and vancomycin in the HF model, corresponding to the dosing regimen of 15 mg/kg of amikacin once a day (A70) and 1 g of vancomycin every 12 h (V18), are provided in **Figure 2**.

For vancomycin, the targeted AUC_24 h_ was 400 μg.h.mL^-1^, i.e., 16.6 times the MIC over 24 h (Toutain et al., 2007), and AUC_24 h_ ranging from 372 to 417 μg.h.mL^-1^, i.e., deviations ranging from -7.0 to +4.3% from the targeted AUC_24 h_, were obtained. For amikacin, the targeted *C*_max_ was 70 μg/mL and, at steady-state, a *C*_max_ of 59.3 ± 25.8 μg/mL (mean ± SD) i.e., a mean deviation of 15.3% from the expected *C*_max_, was obtained.

### PK/PD Study

#### Killing Activity on Planktonic Bacterial Populations

After incubation for 3 days in the HF cartridge (D3), the planktonic and biofilm populations of *S. aureus* were 9.3 ± 0.3 log_10_ CFU/mL and 8.4 ± 0.1 log_10_ CFU/mL, respectively.

**FIGURE 3:**
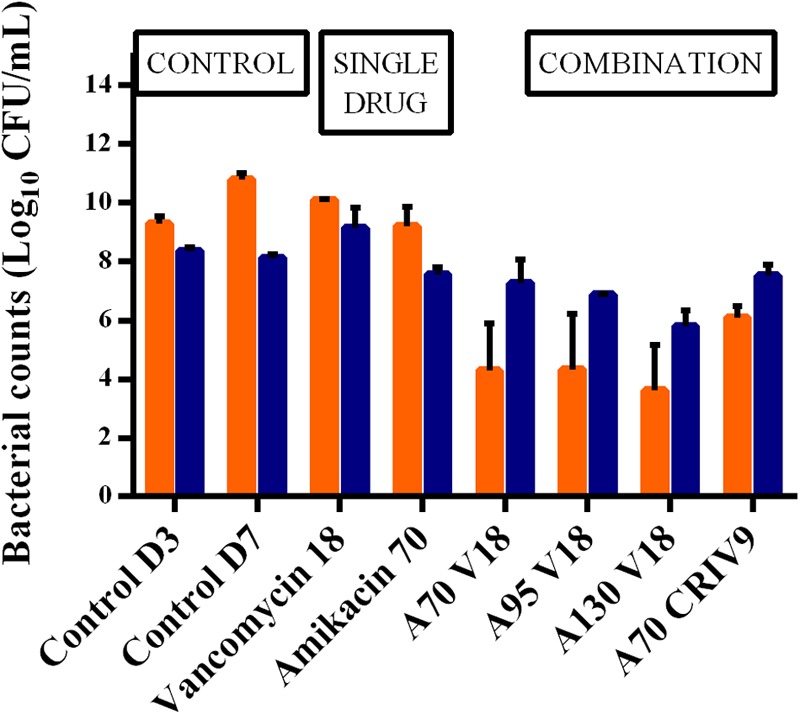
Mean ± SD of the bacterial counts (log_10_ CFU/mL) for planktonic (in orange) and biofilm-embedded bacteria (in blue) at the end of the experiments (D7) for control assays and the different treatments (*n* = 2 for each antibiotic combination). The BEB population was smaller than the planktonic population in the control experiments, and also after monotherapy with amikacin or vancomycin. In contrast, the BEB populations were 1.2–2.0 log_10_ CFU/mL higher than the planktonic populations (*p* < 0.001).

In the absence of antibiotic (control experiments), the planktonic and biofilm populations remained quite stable for a further 5 days with bacterial counts of 10.8 ± 0.2 log_10_ CFU/mL and 8.1 ± 0.1 log_10_ CFU/mL, respectively, at the end of the experiments (D7) (**Figure 3**).

**FIGURE 4:**
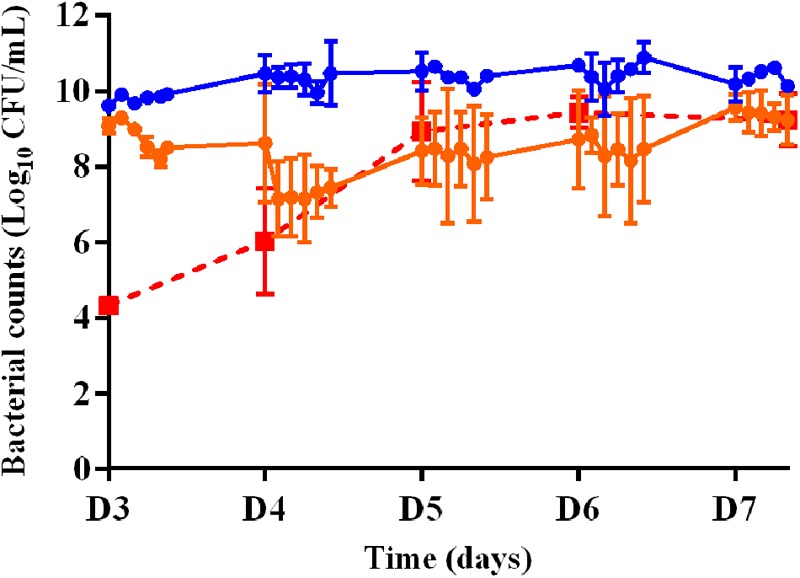
Changes in the planktonic bacterial populations (log_10_ CFU/mL) after exposure to amikacin or vancomycin in monotherapy from D3 to D7. Full circles represent the bacterial counts in the HF model during 5 days of treatment with vancomycin twice a day (V18 treatment, in blue) or amikacin once a day (A70 treatment, in orange). Full red squares represent the bacterial counts of planktonic bacteria growing on agar supplemented with threefold MIC of amikacin over time during A70 treatment. Mean ± SD of the bacterial counts are shown (*n* = 2 for each treatment).

The time-kill curves for the planktonic bacteria associated with the 3-days old biofilm and exposed to amikacin or vancomycin alone and the bacterial counts of planktonic bacteria growing on agar supplemented with threefold MIC of amikacin over time during A70 treatment for 5 days (from D3 to D7) are shown in **Figure 4**. After 5 days of exposure to vancomycin (from D3 to D7) administered twice a day with a peak concentration of 18 μg/mL (V18 treatment), the planktonic population never decreased below the initial population size. After exposure to amikacin administered once a day with a peak concentration of 70 μg/mL (A70 treatment), a mean reduction of 0.9 log_10_ was observed over the 1st day of treatment (D3) but after 5 days (D7), the size of the planktonic population, 9.2 ± 0.7 log_10_ CFU/mL, was very similar to that of the population before exposure to amikacin and not much lower than in the control experiments.

**FIGURE 5:**
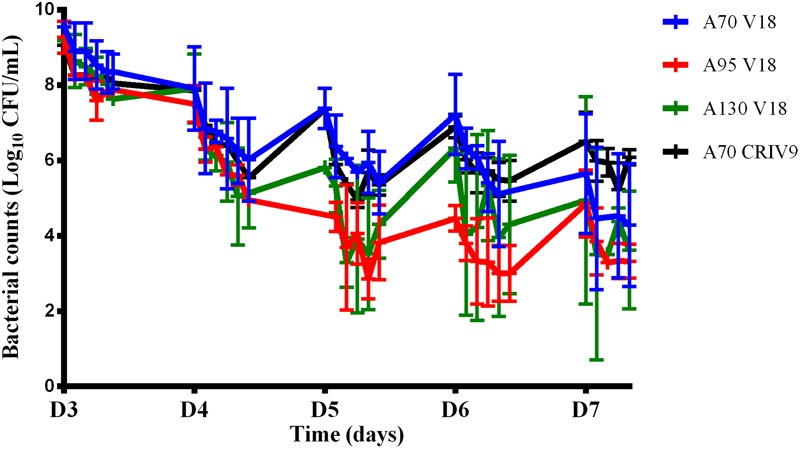
Changes in the planktonic bacterial population (log_10_ CFU/mL) after exposure to combinations of amikacin and vancomycin from D3 to D7. The marks represent the mean ± SD of the bacterial counts for the different tested treatments [blue: A70 V18 treatment, red: A95 V18 treatment, green: A130 V18 treatment and black: A70 CRIV9 treatment (*n* = 2 for each antibiotic combination)]. The reduction of the planktonic bacterial population between the 1st day (D3) and the last day (D7) of treatments with combinations of amikacin and vancomycin was significant (*p* < 0.001).

We then assessed the killing activity of the amikacin and vancomycin combinations over 5 days (from D3 to D7). For amikacin, three peak concentrations of 70 (A70 V18 treatment), 95 (A95 V18 treatment), or 130 (A130 V18 treatment) μg/mL were tested and for vancomycin, a single peak concentration of 18 μg/mL (A70 V18 treatment) twice a day was compared to a steady concentration of 9 μg/mL (A70 CRIV9 treatment). The time-kill curves of planktonic bacteria exposed to the drug combinations from D3 to D7 are shown in **Figure 5**. Similar time-kill profiles were observed for the planktonic bacteria, whatever the drug concentration profiles tested. The mean decrease of the bacterial population during the 1st day of treatment (D3) with the different drug combination regimens was very similar and ranged from -0.9 to -1.4 log_10_ CFU/mL, followed by stabilization or a slight increase overnight. The killing activity of the drugs during the following days (D4–D7) ranged from a decrease of 3.0 log_10_ to an increase of 0.5 log_10_ of the planktonic population between two successive administrations of amikacin.

After exposure to combinations for 5 days (D7), no eradication of planktonic bacteria was observed but the overall reduction ranged from -3.0 log_10_ to -6.0 log_10_ compared to the population before drug exposure. This reduction of the planktonic bacterial population between the 1st day (D3) and the last day (D7) of treatments with combinations of amikacin and vancomycin was significant (*p* < 0.001) whereas amikacin or vancomycin alone failed to reduce the planktonic population over 5 days (the planktonic bacterial populations were equal to or higher after monotherapy than before monotherapy, **Figure 4**).

#### Killing Activity on BEB

The counts of biofilm-embedded bacteria recovered at the end of each experiment (D7) and the planktonic bacterial counts at the same time point are compared in **Figure 3**.

After exposure to vancomycin alone, the BEB count was 9.2 ± 0.7 log_10_ CFU/mL, i.e., approximately one log_10_ higher than the biofilm without treatment, while amikacin alone (A70) decreased the size of the biofilm by 0.6 log_10_ CFU/mL. The addition of vancomycin (V18 or CRI V9) to amikacin (A70) did not increase BEB reduction and showed that the combination did not exhibit any synergy on these bacteria.

In parallel, we observed that the BEB population was smaller than the planktonic population in the control experiments, and also after monotherapy with amikacin or vancomycin. In contrast, the BEB populations were 1.2 to 2.0 log_10_ CFU/mL higher than the planktonic populations (*p* < 0.001) in all the combination experiments.

#### Prevention of the Selection of Resistance

No bacterial growth was observed on vancomycin-supplemented agar, whatever the experiment.

**FIGURE 6:**
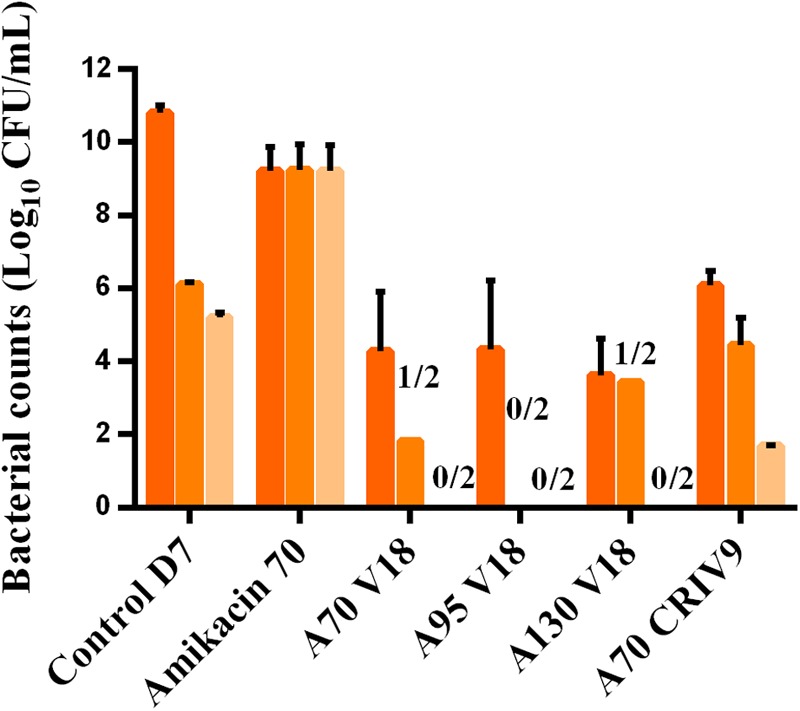
Mean ± SD of the bacterial counts (log_10_ CFU/mL) of total planktonic bacteria (dark orange) and bacteria growing on agar supplemented with three-times the MIC of amikacin (medium orange) and six-times the MIC of amikacin (light orange) in the control experiment or after 5 days of exposure to different treatments (D7) (*n* = 2 for each condition).

**FIGURE 7:**
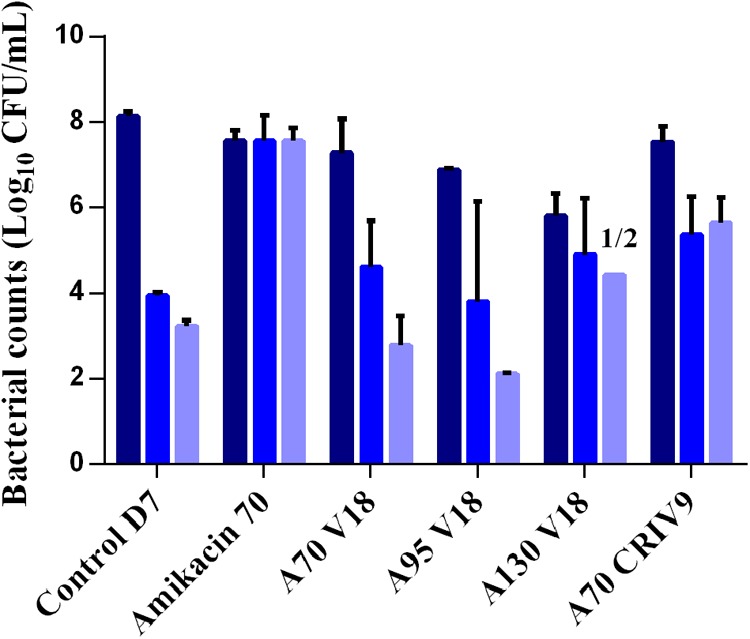
Mean ± SD of the bacterial counts (log_10_ CFU/mL) of total biofilm-embedded bacteria (dark blue) and of biofilm-embedded bacteria growing on agar supplemented with three-times the MIC of amikacin (blue) and six-times the MIC of amikacin (light blue) in the control experiment or after 5 days of exposure to different treatments (D7) (*n* = 2 for each condition).

The counts of planktonic bacteria and BEB growing on agar supplemented with 3-MIC and 6-MIC-amikacin, after exposure to the drugs for 5 days (D7), are compared to the total counts in **Figures 6, 7**. Less-susceptible bacteria were systematically observed on the amikacin- supplemented agar plates before any drug exposure (D3) at a proportion of about 10^-6^ of the total bacterial population for planktonic bacteria (assessed in all the experiments) and BEB (assessed in control experiments). Similar proportions (around 10^-6^) were also found at the end of the control experiments (D7).

After 5 days of exposure to amikacin alone (D7), all the planktonic bacteria and BEB (proportion around 1) were able to grow on 6MIC-amikacin agar (**Figure 6**), which implied that the less-susceptible bacterial population, rather than fully susceptible bacteria, was selected by the drug. The time-development of the less-susceptible planktonic population, represented in **Figure 4**, showed that the fully susceptible population was drastically reduced from the 3rd day of treatment (D5). The addition of vancomycin to amikacin reduced the counts of planktonic bacteria growing on 3-MIC-amikacin and 6-MIC-amikacin, which were only detected in 4 on 1 out of 8 assays, respectively. Exposure of biofilm to the drug combinations, rather than to amikacin alone, also reduced the populations of less-susceptible bacteria (**Figure 7**).

The highest MIC of amikacin for the sampled biofilm bacteria was 16 μg/mL (a 16-fold increase), corresponding to bacteria with intermediate amikacin-susceptibility with regard to the EUCAST breakpoints.

## Discussion

Due to the refractoriness of *S. aureus* biofilm infections to antibiotic treatments, there is an urgent need to optimize the use of currently available drugs to ensure bacterial killing and the prevention of resistance. In this study, we developed an innovative use of the HF model by delaying exposure to the antibiotics and studied the effects of a combination of vancomycin and amikacin both on planktonic bacteria and on BEB in conditions representative of clinical situations. Different concentration profiles of the drugs were tested, and bacteria were subjected to the fluctuating concentrations that might be encountered in patients during a complete treatment. These experimental conditions should have greater predictive value than simple static assays in which bacteria are exposed to a fixed concentration over time. Moreover, due to the lack of medium renewal in static assays, such experiments are often conducted over 24 h whereas longer periods are needed to assess the selection of resistance by antibiotics (Drusano, 2017). Compared to animal models, which may exhibit very different pharmacokinetics to humans and in which some human pathogens cannot develop, all bacteria can be cultured in the HF model and exposed to drug concentration profiles that mimic the range of human profiles (Toutain et al., 2010). For example, as vancomycin is eliminated much faster in mice (half-life = 32 min) than in humans (Knudsen et al., 2000), dosage regimens tested in mice can hardly be extrapolated to humans. Obviously, the main weakness of static or dynamic *in vitro* assays is the absence of the immune system which can cooperate with antibiotics to clear an infection.

Several *in vitro* studies in dynamic systems including the HF model (Nicasio et al., 2012; Lenhard et al., 2016) have investigated the antibacterial activity of drugs combined with vancomycin against planktonic *S. aureus*. However, the use of dynamic *in vitro* systems, such as the CDC biofilm device or others, to study the effects of combinations on biofilm is rarely reported. To our knowledge, the present study is the first to use a HF model to conduct experiments on a 3-day old biofilm of a single *S. aureus* strain to assess the activity of drugs combination, over 5 days, on both planktonic bacteria and BEB. The HF model had already been used to simulate in two distinct studies the free concentration-time profiles of amikacin or vancomycin that can be achieved in patients receiving the recommended doses (Nicasio et al., 2012; Ferro et al., 2015). In our study, exposure to different dosage regimens of a susceptible strain of *S. aureus* with MICs of 1 μg/mL for amikacin and vancomycin led to equal or higher values of the PK/PD indices than those classically expected to obtain drug efficacy (Zelenitsky et al., 2003; Rybak et al., 2009; Song et al., 2015). For aminoglycosides, for which the most predictive PK/PD index is the *C*_max_/MIC ratio (Moore et al., 1987), we targeted *C*_max_/MIC values from 70 to 130 in the HF model whereas a value from 8 to 10 is usually recommended to ensure efficacy against the pathogen (Toutain et al., 2002). For vancomycin, for which the best predictive index is the AUC over 24 h divided by the MIC (AUC_24 h_/MIC) (Nielsen et al., 2011), we targeted the value of 400 recommended to achieve clinical effectiveness (Rybak et al., 2009; Jung et al., 2014; Song et al., 2015) and obtained AUC_24 h_/MIC values ranging from 372 to 417 for the bolus of vancomycin in the HFIM and 480 for the constant infusion. Even though these targeted values of the PK/PD indices were attained for both drugs, almost no bactericidal activity was observed on the 3-day old biofilm or on the co-existing planktonic bacteria when amikacin or vancomycin were administered alone for 5 days. These results are in agreement with previous studies which demonstrated the low activity of vancomycin on large bacterial inocula (LaPlante and Rybak, 2004; LaPlante and Woodmansee, 2009) and on biofilms (Hogan et al., 2016). One study involving a HF model showed that a peak concentration as high as 80 mg/L was needed to achieve bactericidal activity against a large inoculum of a MRSA strain with a MIC of 1 μg/mL for vancomycin (Lenhard et al., 2016). One proposed explanation for the inoculum effect and reduced efficacy of vancomycin is that bacteria at high density are in a stationary growth phase with low dividing rate and low cell wall synthesis (Brown et al., 1988; Lamp et al., 1992). Another explanation is that vancomycin may be sequestrated by *S. aureus* on peptidoglycan layers, thus reducing the free vancomycin concentrations surrounding the bacteria (Srinivasan et al., 2002; Ekdahl et al., 2005; Yanagisawa et al., 2009). Finally, a reduced penetration of vancomycin through *S. aureus* and *S. epidermidis* biofilms has also been described (Doroshenko et al., 2014; Singh et al., 2016) and, even worse than the lack of efficacy, low concentrations of vancomycin were reported to stimulate biofilm formation in some clinical isolates of *S. epidermidis* (Cargill and Upton, 2009). In this study on *S. aureus*, our results were concordant as the biofilm which was exposed to vancomycin alone contained 10 times more bacteria than the control.

The lack of efficacy of the drugs used in monotherapy in this study supports the clinical recommendation to associate an aminoglycoside with vancomycin for the treatment of *S. aureus* biofilm infection (Deresinski, 2009). Compared with the absence of activity of amikacin or vancomycin alone, exposure to combinations of vancomycin and amikacin for 5 days in the HF model had a synergistic bactericidal effect on the planktonic bacterial populations. However, despite this synergy, the planktonic bacteria remaining after 5 days of exposure to the combination (D7) still exceeded 2.5 log_10_ CFU/mL. We therefore investigated the ability of other dosage regimens of amikacin and vancomycin to improve the antibacterial efficacy against this planktonic population. Contrary to our expectations, given the concentration-dependent activity of aminoglycosides, increasing the *C*_max_ of amikacin 1.8-fold (from 70 to 130 μg/mL) did not increase the efficacy on planktonic bacteria. For vancomycin, the efficacy of the combination seemed to be slightly decreased by constant rate infusion, especially on planktonic bacteria, but there were not enough replicates to draw a definitive conclusion. Contrary to the planktonic population, the addition of vancomycin (as a bolus or constant infusion) to amikacin did not result in an additional bacterial reduction on *S. aureus* biofilm, and no synergy between the two drugs was observed. The distinct activity of the combination on planktonic bacteria and BEB confirmed the different phenotypes of these two populations of bacteria and that the drugs were less active on BEB. Indeed, biofilms are supposed to contain more persister bacteria which have lower growth rates and are therefore less affected by antibiotic drugs (Singh et al., 2009; Conlon et al., 2015). Moreover, no dosing regimen tested in this study, even if it exceeded the recommended PK/PD index values, was able to fully eradicate the planktonic bacteria co-existing with a biofilm, which could suggest that some planktonic bacteria were continuously released from the biofilm. As our study is the first one focusing on the biofilm in the HF, microscopy imaging will be further needed to investigate the distribution of the biofilm in the HF cartridge, which could be influenced, among others, by the shear forces in the extracapillary space. It should also be kept in mind that our system was characterized by an absence of the immune system and the presence of a rich medium – more favorable to bacterial growth -, that both limit the efficacy of antibiotic treatments compared to the *in vivo* situation. However, our *in vitro* results are in agreement with the reported lack of efficacy of systemic antibiotic treatments in patients for whom additional treatments, such as mechanical removal of biofilms or very high local antibiotic concentrations, are advised whenever possible (McConoughey et al., 2014; Wu et al., 2015).

In addition to efficacy, we assessed the ability of the combination to reduce the selection of resistant bacteria in planktonic and biofilm populations. The absence of resistance to vancomycin in this study was in accordance with other experiments conducted on *S. aureus* (LaPlante and Rybak, 2004). Conversely, bacteria (approximately 10^-6^) able to grow on agar supplemented with 6 μg/mL (sixfold MIC) of amikacin were systematically present in the planktonic and biofilm populations before drug exposure, implying that small proportions of such bacteria are spontaneously present in large populations, as previously reported (Ferro et al., 2015). Since similar proportions were also found at the end of the control experiments, it suggests that the growth and survival rates of less-susceptible and fully susceptible bacteria were similar in the absence of drugs. After 5 days of antibiotic exposure, the MIC of amikacin for these bacteria able to grow on agar supplemented with amikacin and termed “less-susceptible,” never exceeded the resistance breakpoint (>16 μg/mL). These bacteria showed an intermediate amikacin-susceptibility with regard to the EUCAST breakpoints, implying that the administration of amikacin to patients infected by these bacteria would have an uncertain therapeutic effect (Rodloff et al., 2008), but it should also be stressed that the initial MIC of the tested strain was low (1 μg/mL). This suggests that the same selection phenomenon occurring on a strain with a two or four-fold higher MIC would lead to the selection of “true” resistant bacteria. The selection of less-susceptible bacteria, which represented the main population of planktonic bacteria and BEB after exposure for 5 days to amikacin in monotherapy, could be explained by an inducible mechanism of resistance, known as adaptive resistance, in which thickening of the cell wall results in less penetration of amikacin into the bacterial cell (Yuan et al., 2013). Interestingly, the addition of vancomycin to amikacin considerably reduced the proportions of these less-susceptible bacteria in both planktonic bacteria and BEB compared to amikacin alone, especially when vancomycin was administered in boluses. These results suggest that vancomycin was able to limit the growth of these bacteria less-susceptible to amikacin and prevent their selection. The vancomycin administered by CRI associated with amikacin seemed to limit the selection of less-susceptible bacteria to a lesser extent, but these differences require more thorough investigation.

## Conclusion

By studying planktonic bacteria and BEB in parallel and by mimicking the fluctuations in antibiotic concentrations over 5 days, as can occur *in vivo* after daily administrations, we demonstrated the increased efficacy of a combination of amikacin and vancomycin on planktonic bacteria but not on BEB. However, even though vancomycin did not increase the killing activity of amikacin on BEB, it reduced the selection of bacteria less-susceptible to amikacin, which could help to maintain the efficacy of this drug during treatments. Even if these results need to be further confirmed with clinically relevant strains of MSSA and MRSA, they highlight the importance of selecting combination therapies not only based on efficacy but also on resistance selection endpoints by taking into account the 2 co-existing populations of planktonic bacteria and BEB.

Equations:

(1) $$\text{Concentration}\;\text{HF}\;\text{=}\frac{\begin{array}{l} (\text{Concentration}\;\text{CR}\;\text{*}\;\text{Volume}\;\text{CR})\text{+}\\ \left(\text{Concentration}\;\text{ECS}\;\text{*}\;\text{Volume}\;\text{ECS}\right) \end{array}}{\text{Volume}\;\text{CR+Volume}\;\text{ECS}}$$

With HF being the Hollow-Fiber, CR the Central Reservoir and ECS the Extra-Capillary Space.

## Author Contributions

DB, AF, FW, FE, P-LT, and AB-M: substantial contributions to the conception or design of the work. DB, ML, AF, P-LT, and AB-M: acquisition, analysis, or interpretation of data for the work. DB, ML, FW, FE, P-LT, AB-M, and AF drafting the work or revising it critically for important intellectual content. Final approval of the version to be published. Agreement to be accountable for all aspects of the work in ensuring that questions related to the accuracy or integrity of any part of the work are appropriately investigated and resolved.

## Conflict of Interest Statement

The authors declare that the research was conducted in the absence of any commercial or financial relationships that could be construed as a potential conflict of interest.
